# Neural correlates of cognitive improvements following cognitive remediation in schizophrenia: a systematic review of randomized trials

**DOI:** 10.3402/snp.v6.30054

**Published:** 2016-03-17

**Authors:** Clémence Isaac, Dominique Januel

**Affiliations:** Unité de Recherche Clinique, EPS Ville Evrard, Neuilly-Sur-Marne, France

**Keywords:** cognitive training, cognitive enhancement therapy, neurocognition, social cognition, magnetic resonance imaging, magnetoencephalography, electroencephalography, schizophrenia

## Abstract

**Background:**

Cognitive impairments are a core feature in schizophrenia and are linked to poor social functioning. Numerous studies have shown that cognitive remediation can enhance cognitive and functional abilities in patients with this pathology. The underlying mechanism of these behavioral improvements seems to be related to structural and functional changes in the brain. However, studies on neural correlates of such enhancement remain scarce.

**Objectives:**

We explored the neural correlates of cognitive enhancement following cognitive remediation interventions in schizophrenia and the differential effect between cognitive training and other therapeutic interventions or patients’ usual care.

**Method:**

We searched MEDLINE, PsycInfo, and ScienceDirect databases for studies on cognitive remediation therapy in schizophrenia that used neuroimaging techniques and a randomized design. Search terms included randomized controlled trial, cognitive remediation, cognitive training, rehabilitation, magnetic resonance imaging, positron emission tomography, electroencephalography, magnetoencephalography, near infrared spectroscopy, and diffusion tensor imaging. We selected randomized controlled trials that proposed multiple sessions of cognitive training to adult patients with a schizophrenia spectrum disorder and assessed its efficacy with imaging techniques.

**Results:**

In total, 15 reports involving 19 studies were included in the systematic review. They involved a total of 455 adult patients, 271 of whom received cognitive remediation. Cognitive remediation therapy seems to provide a neurobiological enhancing effect in schizophrenia. After therapy, increased activations are observed in various brain regions mainly in frontal – especially prefrontal – and also in occipital and anterior cingulate regions during working memory and executive tasks. Several studies provide evidence of an improved functional connectivity after cognitive training, suggesting a neuroplastic effect of therapy through mechanisms of functional reorganization. Neurocognitive and social-cognitive training may have a cumulative effect on neural networks involved in social cognition. The variety of proposed programs, imaging tasks, and techniques may explain the heterogeneity of observed neural improvements. Future studies would need to specify the effect of cognitive training depending on those variables.

Schizophrenia is a chronic psychiatric illness characterized by hallucinations, delusions, and thought disorder as well as cognitive and psychosocial impairments. On a neurobiological level, schizophrenic patients suffer from an impaired efficiency of neural processes associated with a decreased functional connectivity (Andreasen, 2010). Cognitive deficits are believed to be a core feature of schizophrenia, observed in patients during the prodromal phase of the illness, mainly in processing speed, working memory, verbal learning, executive functions, and social cognition (Kim et al., 2011). Cognitive impairments are maintained or further increased after the first episode (Becker et al., 2010), but tend to remain stable during the course of illness, thus confirming the hypothesis of a neurodevelopmental rather than a neurodegenerative origin of cognitive deficits in schizophrenia (Lewis, 2004).

Cognitive disorders are estimated to affect approximately 85% of patients. These patients’ cognitive abilities are lower than 90% of healthy subjects (Pfammatter, Junghan, & Brenner, 2006). Cognitive impairments in schizophrenia are related to a poorer psychosocial functioning (McGurk & Mueser, 2004) but are largely independent from psychotic symptoms. Furthermore, studies have observed a stronger correlation between cognitive functioning and social functioning than between symptoms and social functioning (Penadés et al., 2010). Stabilized schizophrenia patients often suffer from cognitive impairments, and antipsychotic treatments have a limited impact on cognition (Harvey, 2009) and psychosocial functioning (Hegarty, Baldessarini, & Tohen, 1994). Despite these observations, psychotic symptoms are the main target of treatments proposed to patients suffering from schizophrenia.

McClure et al. (2007) have shown that processing speed, episodic memory, and executive functions are specifically correlated with autonomy in daily life in schizophrenia; on the other hand, social skills are more strongly associated with working memory and verbal fluency. Given the relationship between cognition and psychosocial functioning, as well as the limited impact of pharmacotherapy on these features, cognitive training seems a promising tool for patients suffering from schizophrenia. Indeed, patients and their relatives seek treatments focusing on improving functioning and quality of life, rather than psychotic symptoms only (Marder, 2008).

Cognitive remediation (CR) is a type of psychosocial care consisting of cognitive functions training, in the form of series of exercises of progressive difficulty (Medalia & Choi, 2009). Its objective is to promote patients’ autonomy, functional recovery, and resistance to environmental stressors, through the reduction of their cognitive deficits (Dubeau, Salomé, & Petitjean, 2007). The neuroplasticity model underlying CR hypothesizes the development of functional compensation mechanisms by the recruitment of neural areas previously specialized in other functions (Bottéro, 2010). Research in the 1970s has shown that learning and memory strategies can improve dysfunctional cognitive abilities in schizophrenia (Passerieux & Bazin, 2009).

Multiple studies and meta-analysis reported CR's efficacy on the cognitive and functional levels (McGurk, Twamley, Sitzer, McHugo, & Mueser, 2007; Penadés et al., 2010; Tomás, Fuentes, Roder, & Ruiz, 2010). Indeed, the stability of neuropsychological measures in schizophrenia over a period of up to 10 years suggests that the observed improvements following CR can be related to this intervention and not to the natural course of illness (Wykes & Spaulding, 2011). CR has yielded promising results with desk-work or computerized exercises, in individual or group sessions, with persisting effects up to 8 months after the end of therapy, especially in verbal memory, working memory, and executive functions (McGurk et al., 2007; Wykes et al., 2003). However, the effect of CR on cognitive functioning depends largely on the programs’ cognitive targets. These targets may be the most frequently altered cognitive functions in schizophrenia, those recognized as having functional repercussions, or those which are observed in a particular patient (Reeder, Newton, Frangou, & Wykes, 2004). Furthermore, the results reported by different studies show a great heterogeneity, depending on patients and used programs. Future studies comparing different programs and treatment modalities might be able to determine the active ingredients that can potentiate CR in schizophrenia (Tomás et al., 2010).

Neuroimaging studies have been conducted during the past few years in order to specify the efficacy of CR on neural processes and the relationship with behavioral and psychosocial improvements. A recent meta-analysis investigated the effect of working memory training in schizophrenia and healthy subjects (Li et al., 2015). Authors reported a possible neuroplastic effect of working memory training in schizophrenia, mainly in the dorsolateral prefrontal cortex (DLPFC), the precuneus, and the fusiform gyrus. Another meta-analysis observed the neurobiological effect of CR in schizophrenia in functional magnetic resonance imaging (fMRI) studies (Ramsay & MacDonald, 2015). They observed an effect of CR on various cortical and subcortical areas known to support cognition and psychosocial functioning. Interestingly, they observed no relationship between the level of improvement after therapy and the different modalities of CR such as training intensity, approach, or used material (desk-work or computerized exercises). These meta-analyses provide partial conclusions on the neurobiological effects of CR, on working memory only, or as assessed with fMRI only. Moreover, a qualitative review addressed the question of the efficacy of CR on neural processes (Thorsen, Johansson, & Løberg, 2014) and concluded that there was an effect of CR on neural activity and structure, mainly in prefrontal regions, but also in parietal and limbic areas. However, several studies in that review were not randomized controlled studies, and the question remains as to whether specific changes associated with CR in schizophrenia differ from those found following other therapeutic interventions or usual psychiatric care.

Our objective was to explore the neural substrates of cognitive enhancement following CR interventions in schizophrenia. To do so, we reviewed randomized controlled trials that proposed a cognitive training program to adult patients suffering from schizophrenia, comparing this intervention to either a control therapy or treatment as usual (TAU), and assessing neural processes through imaging techniques, with or without neuropsychological testing to assess cognition.

## Method

We conducted a systematic search of online databases from May 2015 until July 2015. Databases included MEDLINE, PsycInfo, and ScienceDirect. Additional studies were identified through a manual reference search. In order to conduct a systematic review, we followed the Preferred Reporting Items for Systematic reviews and Meta-Analyses (PRISMA) guidelines (Moher, Liberati, Tetzlaff, Altman, & The PRISMA Group, 2009).

### Eligibility criteria

We selected randomized controlled trials that assessed the effect of CR interventions for schizophrenia using imaging techniques.

Prior to report searching, we determined the criteria for a study to be eligible for our review. Concerning our population, the study needed to include adult patients suffering from a schizophrenia spectrum disorder. The choice for schizophrenia spectrum disorders instead of schizophrenia only is because a large number of clinical trials on CR include patients without differentiation between schizophrenia, schizoaffective disorder, and schizophreniform disorder (McGurk et al., 2007). Including studies on schizophrenia spectrum disorders in this review may help to provide useful conclusions for health care providers. Concerning the intervention, we only included studies where patients were provided with multiple sessions of cognitive training. Training of neurocognition or social cognition was considered eligible. However, studies evaluating the effect of CR on social cognition will be discussed independently from those evaluating its effect on neurocognition. We selected studies where any imaging technique was used to assess the effect of cognitive training. Assessments needed to be conducted at baseline and right after therapy. We included randomized studies that compared patients receiving a cognitive training and patients receiving another therapeutic intervention or TAU.

We determined criteria for the eligibility of scanned reports. First, we excluded all articles that did not involve any study (e.g. review, letter to the editor) or that involved studies without results (e.g. presentation of a CR program or a study design). Moreover, we restricted our selection to peer-reviewed written communications such as articles and doctoral theses, excluding poster presentations, oral communications, and chapters of books. No publication date or language restrictions were applied, provided that an abstract in English was available.

### Search and selection methods

Search terms were selected to target our population (schizophrenia), intervention (CR, cognitive training, rehabilitation), and assessment technique (magnetic resonance imaging, diffusion tensor imaging [DTI], positron emission tomography, near infrared spectroscopy, electroencephalography [EEG], magnetoencephalography [MEG]) of interest (see Appendix for search method for the MEDLINE database).

After database searching, reports where scanned according to their authors, title, and Digital Object Identifier (DOI) if available, in order to remove duplicates. Reports were then screened by one of the authors (CI) on the basis of their title and abstract and were excluded if they clearly didn't meet the eligibility criteria cited above. Remaining reports were screened by the same author (CI) on the basis of their full text, using a form to identify precisely if they met eligibility criteria. When required by a specific report, independent screening by another author (DJ) and consensus between the two authors in case of disagreement were applied. Additional reports were then identified through manual search (online search, search in books, included studies’ literature search). Finally, multiple publications were identified and combined to avoid biases. Studies were recognized as multiple publications when two or more publications used the same imaging technique for the same sample of patients and the same intervention.

### Data collection process

For each included study, a form was completed to obtain information on study design, population, intervention, assessments, results, and risk of bias. Risk of bias was assessed by adapting Cochrane risk of bias tool (Higgins, Altman, & Sterne, 2011) and including it in our form. We contacted three authors for further information. All responded and provided details on inclusion criteria, study design, and risk of biases.

Because of the heterogeneity of methodology, population, intervention, and assessment, we've chosen a qualitative description of the included studies rather than a quantitative synthesis.

## Results

### Studies selection

**Fig. 1:**
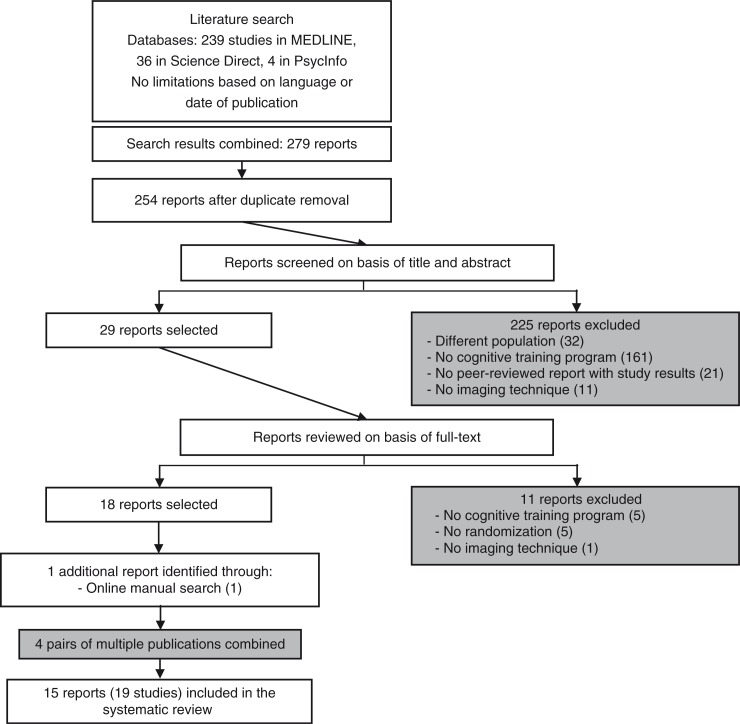
Publication selection process.

The search on MEDLINE, PsycInfo, and ScienceDirect databases provided 279 citations. Two hundred fifty-four reports remained after removing duplicate citations. We excluded 225 reports after screening their title and abstract because they clearly didn't meet our eligibility criteria: 32 reports had a different population, 161 of the remaining reports didn't propose a CR program, 21 of the remaining reports were not peer-reviewed or didn't present study results, and 11 of the remaining reports didn't use imaging techniques. Of the 29 remaining reports reviewed on the basis of their full text, 11 were excluded because they didn't meet our criteria for intervention (five reports), study design (five reports), or assessment (one report). An additional report was found through online manual search. Finally, four pairs of reports were combined because they were identified as multiple publications. Multiple publications were explicitly stated as so in the reports or by the authors we contacted. In total, 15 reports involving 19 studies were included in the systematic review (see Fig. 1 for a diagram summarizing the selection process).

### Studies’ characteristics

Table 1 and 2 show the characteristics of included studies and main results. Included reports involved a total of 455 adult patients: 271 received CR, 111 benefited from another intervention, and 73 received TAU. Patients suffered from a schizophrenia spectrum disorder (nine reports) or specifically from schizophrenia (six reports).

**Table 1:** Effect of cognitive remediation on neurocognition

Authors	Subjects^a^	Groups	Target CR characteristics	Technique and task	Significant neurobiological results^b^	Significant behavioral improvements^b^	Positive significant correlations^b^
*Magnetic resonance imaging*							
Bor et al., 2011	17 SCZ 15HV	Rehacom (8) vs. TAU(9)	14 computerized sessions	fMRI: verbal/spatial n-back *T0; T1 (3 months)*	Rehacom>TAU (T1-T0) for spatial n-back: ↑ left middle and inferior frontal gyri, cingulate gyrus, inferior parietal lobule	Rehacom>TAU (T1-T0): attention and reasoning	↑ Broca's area ***COR*** ↑ attention
Eack et al., 2010	53 SCS	CR(30) vs. supportive therapy(23)	60 computerized+45 group sessions (neuro and social cognition)	VBM *T0; T1 (1 year); T2 (2 years)*	CR (T2-T1; T1-T0): – ↓ gray matter loss: left medial temporal; anterior cingulate gyrus – ↑ gray matter: left amygdala	CR>control (T2): verbal memory, executive functions and social cognition (Eack et al., 2009)	↑ behavioral ***COR*** ↓ gray matter loss in left parahippocampal and fusiform gyrus ↑ Social cognition ***COR*** ↑ gray matter in left amygdala.
Haut et al., 2010	18 SCS 9 HV	CR(9) vs. CBT(9)	25 computerized group sessions	fMRI: word/picture n-back, lexical decision *T0; T1 (8 weeks)*	CR>control (T1-T0) for n-back: ↑ prefrontal cortex, anterior cingulate gyrus	–	↑ activations ***COR*** ↑ n-back
Penadés et al., 2013	30 SCZ 15 HV	CRT(15) vs. SST(15)	40 desk-work sessions	DTI; fMRI: number n-back *T0; T1 (4 months)*	CRT (T1-T0): – ↑ functional anisotropy in genu and body of the corpus callosum and posterior thalamic radiation – ↓ central executive network (middle and inferior frontal and anterior cingulate gyri, inferior and superior parietal lobe, precuneus) – ↓ default mode network (anterior cingulate, cingulate left middle temporal and left supramarginal gyri, precuneus, cuneus)	CRT>SST (T1): categorization, planning, memory	↓ central executive network ***COR*** ↑ functional anisotropy in the corpus callosum and ↑ cognition
Subramaniam et al., 2012, 2014	29 SCZ 12 HV	CR(15) vs. Computer games(14)	80 computerized sessions (auditory; visual; social cognition)	fMRI: reality-monitoring task *T0; T1 (16 weeks)*	CR>control (T1) for self vs. externally-generated words: ↑ medial prefrontal cortex	CR>control (T1): self-generated words accuracy CR (T1-T0): delayed memory, executive functions	Medial prefrontal cortex activation (T1) ***COR*** ↑ verbal memory and self-generated words accuracy
							
				fMRI: letter n-back *T0; T1 (16 weeks)*	CR>control (T1-T0): ↑ left middle and inferior frontal gyri	CR>control (T1-T0): n-back CR (6 months-T1): working memory	↑ right middle frontal gyrus ***COR*** ↑ 2-back and ↑ left inferior and left middle frontal gyri
Vianin et al., 2014	16 SCZ	RECOS (8) vs. TAU (8)	28 computerized sessions (executive function)	fMRI: verbal fluency task *T0; T1 (6 months)*	RECOS>TAU (T1): ↑ right precentral and left inferior frontal gyrus, left middle cingulate cortex	RECOS (T1-T0): inhibition and reasoning	–
Wykes et al., 2002	12 SCZ 6 HV	CRT(6) vs. occupational therapy (6)	40 desk-work sessions	fMRI: letter n-back *T0; T1 (3 months)*	CRT>control (T1): ↑ right inferior frontal gyrus, occipital cortex	CRT>control (T1-T0): memory	–
*Electroencephalography*							
Kariofillis et al., 2014	46 SCS	AUD(16) vs. VIS(15) vs. TAU(15)	10 group computerized sessions (auditory)	ERP: oddball *T0; T1 (2 weeks); T2 (2 months)*	↓ P2 latency for AUD (FCz; Cz) and VIS (Fz; FCz); ↓ N1 latency; ↑ N2 amplitude; (T1-T0)	AUD, VIS: working memory	No correlations
Rass et al., 2012	44 SCS	CR(17) vs. watching videos(17) vs. TAU (10)	20 computerized sessions	ASSR; ERP: oddball *T0; T1 (5 weeks); T2 (10 weeks); T3 (20 weeks)*	CR, TAU: ↓ P3 amplitude over time No ASSR effect.	All groups: processing, memory CR>control (T3-T2): visuospatial memory	–
*Magnetoencephalography*							
Dale et al., 2016	33 SCS 13 HV	AT(17) vs. Computer games(16)	50 computerized sessions (auditory)	Auditory discrimination task *T0; T1 (10 weeks)*	AT (T1-T0): ↑ M100; ↑ gamma in left frontal then bilateral temporal	AT (T1-T0): verbal memory	AT: ↑ M100 ***COR*** ↑ executive function
Popov et al., 2011, 2012	39 SCS 15 HV	CE(20) vs. Cogpack (19)	20 computerized sessions (auditory)	Passive hearing of paired clicks *T0; T1 (4 weeks)*	CE>Cogpack: ↓ M50 gating ratio at onset of the second click	CE>Cogpack (T1-T0): verbal short-term and working memory	CE: ↓ of gating ratio in the left hemisphere ***COR*** ↑ short-term verbal memory ↓ of gating ratio in the right hemisphere ***COR*** verbal working memory.
							
					CE (T1-T0): ↑ gamma in a centro-parietal cluster		CE: ↓ alpha in a centro-parietal cluster ***COR*** ↑ verbal memory
					CE: ↓ alpha in bilateral temporal and central clusters Cogpack: ↓ alpha in a left temporal cluster		

SCZ: schizophrenia; HV: healthy volunteers; SCS: schizophrenia spectrum disorders; TAU: treatment as usual; CR: cognitive remediation; CBT: cognitive behavioral therapy; RECOS: cognitive remediation for patients with a schizophrenia spectrum disease; CRT: cognitive remediation therapy; SST: social skills training; AUD: auditory cognitive training; VIS: visuo-spatial cognitive training; AT: auditory training; CE: auditory-focused cognitive exercises; fMRI: functional magnetic resonance imaging; VBM: voxel-based morphometry; DTI: diffusion tensor imaging; ERP: event-related potentials; ASSR: auditory steady state response; MEG: magnetoencephalography.

a Subjects for whom neurobiological data were analyzed

b Results focus on patient samples and not healthy volunteers.

↑ Increased response or performance; ↓ decreased response or performance; ***COR*** significant positive correlation.

**Table 2:** Effect of cognitive remediation on social cognition

Authors	Subjects^a^	Groups	target CR characteristics	Technique and task	Significant neurobiological results^b^	Significant behavioral improvements^b^	Positive significant correlations^b^
*functional Magnetic Resonance Imaging*							
Habel et al., 2010	20 SCZ 10 HV	TAR(10) vs. TAU(10)	12 computerized and desk-work group sessions (affect recognition)	Facial affect recognition; facial age discrimination *T0; T1 (6 weeks)*	TAR>TAU (T1-T0) for affect recognition: ↑ inferior frontal, left occipital and right parietal regions TAR>TAU (T1-T0) for age discrimination: ↑ superior temporal, precentral, postcentral, posterior cingulate and parahippocampal gyri, thalamus, basal ganglia	TAR: affect recognition accuracy	TAR: ↑ affect recognition accuracy ***COR*** ↑ occipital, inferior parietal, inferior and middle frontal gyrus

Hooker et al., 2012, 2013	22 SCS	AT+SCT (11) vs. Computer games(11)	50 computerized sessions (auditory and social cognition training)	Facial affect recognition; object color recognition *T0; T1 (10 weeks)*	AT+SCT (T1-T0): – emotions vs. objects: ↑ right postcentral gyrus – negative vs. objects: ↓ gyrus rectus, medial superior frontal gyrus – positive vs. negative: ↑ globus pallidus	AT+SCT>control (T1-T0): recognition of emotion in faces and scenes, identification of positive emotions	↑ emotion perception ***COR*** ↑ postcentral for emotions vs. object ↑ emotion perception ***COR*** ↑ left angular for positive vs. object ↑ emotion perception ***COR*** ↑ left precentral for positive vs. negative
							
				Recognition of emotions on morphed faces *T0; T1 (10 weeks)*	AT+SCT>control (T1-T0) for open-mouth emotions: ↑ amygdala		↑ emotion perception ***COR*** ↑ right amygdala, putamen and medial prefrontal for open-mouth emotions

*Electroencephalography*							
Luckhaus et al., 2013	15 SCS	Cross-over: TAR(9) vs. Waiting list+TAR(6)	12 computerized and desk-work group sessions (affect recognition)	ERP: facial affect recognition *T0; T1 (2 months); T2 (4 months)*	No change in ERP after TAR. sLORETA analysis: – early ↓ : left inferior parietal, temporal lobe – later ↑ : right superior and middle frontal gyri, anterior cingulate gyrus	TAR (T1-T0): facial affect recognition accuracy (maintained at T2)	–
*Magnetoencephalography*							
Popov et al., 2015; Popova et al., 2014	61 SCS	FAT(20) vs. CE(20) vs. TAU(21)	20 computerized sessions (affect recognition)	Hearing of paired clicks *T0; T1 (4 weeks)*	CE (T1-T0): ↓ Alpha power at onset of second click	All groups (T1-T0): processing speed, attention, visual learning CE (T1-T0): reasoning	CE: ↓ alpha power ***COR*** ↑ verbal learning
							
				Watching of morphing emotions on faces *T0; T1 (4 weeks)*	FAT>CE, FAT>TAU (T1): ↑ alpha power during task pre-morphing period		FAT: ↑ alpha power in fronto-central region ***COR*** ↑ blended emotions task

SCZ: schizophrenia; HV: healthy volunteers; SCS: schizophrenia spectrum disorders; TAR: treatment of affect recognition; TAU: treatment as usual; AT: auditory training; SCT: social cognition training; FAT: facial affect recognition training; CE: auditory-focused cognitive exercises; fMRI: functional magnetic resonance imaging; ERP: event-related potentials; MEG: magnetoencephalography; sLORETA: standardized low resolution brain electromagnetic tomography.

a Patients that received CR and for whom neurobiological data were analyzed

b Results focus on patient samples and not healthy volunteers.

↑ Increased response or performance; ↓ decreased response or performance; ***COR*** significant positive correlation.

Nine reports concerned programs training neurocognition, three used social cognition training programs, and three combined remediation of social and non-social cognition. The number of sessions varied between 10 (Kariofillis, Sartory, Kärgel, & Müller, 2014) and more than 100 (Eack et al., 2010).

All 15 selected reports were randomized trials published in English between 2002 and 2015. All but one used a parallel-arm design and the remaining one used a cross-over design. Eight reports compared the active treatment to a positive-control group, four compared the active treatment to TAU, and three compared the active treatment both to a positive-control group and to TAU. Two reports did not include neuropsychological assessments.

Neural processes were explored with magnetic resonance imaging (nine reports), MEG (three reports), or EEG (three reports).

### Studies’ results

Table 1 shows a summary of included studies observing the effect of CR on neurocognition. Concerning functional neuroimaging trials, six studies observed a significant greater increase in activation of frontal regions after CR (Bor et al., 2011; Haut, Lim, & MacDonald, 2010; Subramaniam et al., 2012, 2014; Vianin et al., 2014; Wykes et al., 2002), including the prefrontal cortex (Haut et al., 2010; Subramaniam et al., 2012), and three of them found an improvement in cingulate gyrus activation (Bor et al., 2011; Haut et al., 2010; Vianin et al., 2014). Increased activations were also observed in parietal (Bor et al., 2011) and occipital (Wykes et al., 2002) regions. On the contrary, one study reported a decreased activation in regions of the central executive network that were overactivated at baseline and in regions associated with the default mode network (Penadés et al., 2013). Concerning structural neuroimaging studies, a trial using DTI observed an increased functional anisotropy of the genu of the corpus callosum after CR (Penadés et al., 2013). A voxel-based morphometry longitudinal study found a neuroprotective effect of CR on gray matter loss, with significantly less gray matter loss for patients that received CR in left fusiform gyrus, left hippocampus, and left parahippocampal gyrus, and a significant gray matter growth in left amygdala (Eack et al., 2010). Furthermore, one report found a correlation between functional and structural improvements (Penadés et al., 2013) and another observed a correlation between improvements in left and right frontal activations (Subramaniam et al., 2014).

Two EEG studies assessed CR effects using an oddball task, an auditory selective attention task used to compare the neural response associated with the processing of repetitive or deviant stimuli. One of them found a decreased P3 amplitude over time, an event-related potential associated to the intensity of attentional resources being engaged (Rass et al., 2012). The other study didn't observe such results but found a decreased P2 latency, associated with working memory, in fronto-central regions after training (Kariofillis et al., 2014). Two studies observed a change in oscillatory activity with MEG, with an increased gamma activity after therapy (Dale et al., 2016; Popov, Rockstroh, Weisz, Elbert, & Miller, 2012), correlated for one study with a decrease in alpha response (Popov et al., 2012). Moreover, one study found an increased M100 response, related to auditory processing, after training (Dale et al., 2016), whereas another only observed a positive effect of CR on M50, associated with the inhibition of redundant stimuli (Popov et al., 2011).

Only four reports observed the neurobiological effect of CR on social cognition (See Table 2 for a summary of included studies). Concerning fMRI studies, improvements were observed after therapy mainly in frontal and parietal regions (Habel et al., 2010; Hooker et al., 2012) and in limbic structures (Habel et al., 2010; Hooker et al., 2012). Only one EEG study observed the effect of social cognition training and found an early activation decrease in left inferior parietal and temporal lobes and a later activation increase in right superior and middle frontal gyri and anterior cingulate cortex after therapy (Luckhaus, Frommann, Stroth, Brinkmeyer, & Wölwer, 2013). Only one MEG report investigated the effect of social cognition training and observed a decreased alpha power in an auditory task and an increased alpha power in a facial emotion task after CR (Popov et al., 2015; Popova et al., 2014).

Overall, 11 reports provided results on correlations between neuropsychological and neurobiological data, and all but one (Kariofillis et al., 2014) found a significant positive correlation between improvements in behavioral and neurobiological results.

Eight reports assessed symptomatology before and after therapy. Two reports observed no correlation between the evolution of symptoms and behavioral results (Haut et al., 2010) or fMRI results (Subramaniam et al., 2012, 2014). Five reports observed an improvement in clinical symptoms without significant difference between CR and control group (Bor et al., 2011; Habel et al., 2010; Kariofillis et al., 2014; Popov et al., 2011, 2012, 2015; Popova et al., 2014) and one report observed no clinical change throughout the study in either group (Luckhaus et al., 2013).

Only 6 out of 15 reports provided results on functional outcomes. Four reports used the Global Assessment of Functioning scale, and found either no correlation between functional and behavioral improvements on a working memory task (Haut et al., 2010) or an improvement in functioning with no significant difference between the CR and control conditions (Kariofillis et al., 2014; Popov et al., 2011, 2012, 2015; Popova et al., 2014). Furthermore, two reports used the Quality of Life scale and observed no improvement in quality of life in either group (Hooker et al., 2012, 2013; Subramaniam et al., 2012, 2014). However, one report found a correlation between neural activation after CR and quality of life at a 6-month follow-up (Subramaniam et al., 2012, 2014).

Only three reports provided follow-up data on neural processes. One report did not find a significant effect of CR 6 weeks after a 2-week program (Kariofillis et al., 2014), whereas a report observed a maintained improvement 2 months after a facial affect training 2-month program (Luckhaus et al., 2013) and another found a significant improvement in the CR group in visuospatial memory 10 weeks after a 10-week program, when compared to an active control group (Rass et al., 2012).

### Risk of bias

Table 3 shows a summary of the risk of bias within reports. Of the 15 included reports, 2 had only partial allocation concealment. One did not randomize three patients that were familiar with the CR program and were used as positive control (Popov et al., 2011, 2012). These patients were assigned to the group receiving the intervention that was hypothesized to be the most effective for auditory processing assessed in these studies. This partial randomization may have biased results, as at least 3 of the 20 patients in the active group had a previous experience of CR. Another study added the control TAU group after the beginning of the study, when 10 patients were already randomized in the active or positive-control groups (Rass et al., 2012). Hence, assessors were not blinded for a subsample in said study, possibly biasing results concerning the effect of the two treatments as compared to TAU.

**Table 3:** Risk of bias

Authors	Full-sample randomization	Blinding: participants^a^	Blinding: assessors^a^	Drop out	Incomplete patient data	Selective outcome reporting	Data substitution	Payment^a^	Conflict of interest
Bor et al., 2011	Yes	No	Yes	15%	NR	No	NR	Yes	NR
Dale et al., 2016	Yes	Yes	Yes	NR	18% 3 patients without NPD	No	NR	Yes	One author (consultant for the CR program company)
Eack et al., 2010	Yes	No	No	13% (33% at 1 year; 37% at 2 years)	7%	No	using maximum-likelihood estimation	No	Two authors (developed the CR program)
Habel et al., 2010	Yes	No	No	NR	6 patients without affect recognition and 7 without age discrimination behavioral data	No	NR	No	One author (developed the CR program)
Haut et al., 2010	Yes	No	–	16%	12%	No	NR	No	NR
Hooker et al., 2012, 2013	Yes	Yes	Yes	14%	7%	No	subject pre to post/post to pre data	Yes	Two authors (consultants for the CR program company)
Kariofillis et al., 2014	Yes	No	Yes	NR	3 patients without follow-up	No	NR	Yes	NR
Luckhaus et al., 2013	Yes	No	–	21%	NR	No correlation between behavioral and NBD	for group mean	No	Two authors (developed the CR program)
Penadés et al., 2013	Yes	No	Yes	NR	14%	No	NR	No	NR
Popov et al., 2011, 2012	3 patients not randomized	Yes	Yes	22%	6% for alpha/gamma analysis 4 patients without NPD	No	NR	Yes	One author (developed the CR program and executive officer of the company)
Popov et al., 2015; Popova et al., 2014	Yes	Yes	No	24%	5%	No	NR	Yes	NR
Rass et al., 2012	10 subjects not randomized in TAU	No	Yes	13% before the end of therapy but still assessed	8%	No correlation between NPD and NBD	NR	Yes	NR
Subramaniam et al., 2012, 2014	Yes	Yes	Yes	3%	3% for reality-monitoring, 6% for n-back 4 patients without follow-up	Missing data on correlation between NPD and NBD	NR	Yes	Two authors (work for the CR program company)
Vianin et al., 2014	Yes	No	Yes	NR	NR	No correlation between NPD and NBD	NR	No	One author (developed the CR program)
Wykes et al., 2002	Yes	No	No	NR	5 patients without complete NPD	No correlation between NPD and NBD	NR	No	One author (developed the CR program)

TAU: treatment as usual; NR: none reported; NPD: neuropsychological data; NBD: neurobiological data; CR: cognitive remediation.

a Payment and blinding were assumed to be non-existent if not specified in the report or online study record.

Eleven reports compared the active group to a positive control but to our knowledge, only four of them reported a double blind design. In five reports neither assessors nor patients were blinded, although in two of them patients didn't receive any neuropsychological assessment. Assessors only were blind in five reports, and one report mentioned blinding of participants only. Partial or no blinding in a majority of the included studies might have affected results, through patients or researchers’ biased attitudes and expectations (Schulz & Grimes, 2002).

Six publications did not report any drop out. This data may not entirely reflect a high compliance with treatment, but rather seems to be because several of these studies include a subsample of patients that received a neurobiological assessment, within a larger randomized trial on CR. Other publications reported a drop-out rate of between 3 and 24%, except for Eack et al. (2010) that had the longest CR duration and reported a drop-out rate of 13% before the second assessment, 33% at 1 year, and 37% at 2 years of intervention. To our knowledge, the rate of incomplete neurobiological data in included reports goes from 0 to 18%. There was no particular difference in incomplete data rate depending on the technique used to assess neural processes. As stated above, four reports did not provide results on correlations between behavioral and neurobiological data. An additional report provided incomplete correlations between neuropsychological and neurobiological results, because of the use of multiple publications reporting different neuropsychological results (Subramaniam et al., 2012, 2014). Moreover, one report assessed patients that had ended therapy before the end of the 20 sessions (Rass et al., 2012). Finally, three reports used missing data substitution methods in order to maintain statistical power.

Patients received remuneration in 8 of the 15 reports. Receiving financial compensation for participation in a study may bias recruitment or affect intrinsic motivation of patients during the intervention, therefore modifying results. Finally, conflict of interest might have occurred in four reports where four different authors had a financial interest in the company that sells the CR program of interest in said studies.

## Discussion

### Remediation of neurocognition

#### Evidence of normalized cerebral activations following CR

Several studies reported a neuroplastic effect of CR when compared to another intervention or TAU, with mainly frontal but also occipital cortex and anterior cingulate gyrus increased activations during working memory and executive tasks. Five reports used an n-back working memory task with a 0-back attentional control condition and a 2-back condition that targets working memory. The first published report in 2002 compared 40 desk-work CR sessions to occupational therapy (Wykes et al., 2002). Authors observed an increased activation in the right inferior frontal gyrus and the right occipital cortex during an untrained fMRI verbal 2-back task. Patients that improved their performance in memory had a significantly greater improvement in activation when compared to non-improved patients. Authors hypothesized that the change in occipital cortex activation was due to an increased use of visual processing strategies during the verbal task (Wykes et al., 2002).

Subramaniam et al. (2014) found a similar effect after 80 computerized sessions of cognitive training targeting both social and non-social cognition, with a significantly greater signal increase after therapy compared to an active computer games playing control group, in left middle and inferior frontal gyri during an untrained letter n-back task. Interestingly, authors also observed an effect of therapy on a reality-monitoring task. In this task, patients had to recall if words semantically related to a prime had been previously externally-presented or self-generated by the patient. After therapy, authors observed a significant increase in medial prefrontal cortex activation for correct self-generated trials compared to externally-presented trials. This increased activation was positively correlated after therapy with an improved reality-monitoring accuracy for self-generated words and an improved delayed verbal memory recall. Furthermore, increased activation was correlated to an improved social functioning six months after therapy. Increased frontal activation after CR could be the result of an intensive training in verbal memory coupled with the learning of self-monitoring strategies (Subramaniam et al., 2012; Wykes et al., 2002).

Other authors observed a significant increase in activation in anterior cingulate gyrus, prefrontal areas (Haut et al., 2010), and Broca's area (Bor et al., 2011) during a spatial n-back task after CR. A similar increased frontal activation, and especially Broca's area, during a covert verbal fluency task, was found in another study comparing 28 biweekly computerized CR sessions focusing on executive functions to TAU in 16 patients suffering from schizophrenia (Vianin et al., 2014). Increased activation during these tasks could be explained by the use of verbal strategies during training (Bor et al., 2011; Vianin et al., 2014). One of CR's strategies consists of helping the patient to use multimodal information processing. This could explain an increased Broca's area activation during visual working memory and covert verbal fluency tasks and an improvement in visual areas for verbal tasks (Bor et al., 2011; Vianin et al., 2014; Wykes et al., 2002). Moreover, prefrontal cortex and anterior cingulate gyrus are involved in two processes frequently impaired in schizophrenia, working memory maintenance, and executive control (Kerns et al., 2005). Hence, these results could indicate that patients used more complex executive strategies during the tasks after therapy (Haut et al., 2010).

These results are in accordance with a study that observed a significantly increased brain-derived neurotrophic factor after 2 weeks of CR, normalized to the level of healthy subjects after 10 weeks of treatment (Vinogradov et al., 2009). Furthermore, present results partially replicate existing findings on the behavioral effect of CR. Multiple studies reported a behavioral effect of remediation programs promoting the development of problem solving strategies, especially on memory functions. These programs can improve memory in schizophrenia patients to the level of healthy controls (McGurk et al., 2007). Wykes et al. (2003) suggested that neuropsychological memory improvement after therapy parallels modification in storage and recall strategies.

Moreover, a longitudinal study using voxel-based morphometry provided evidence of a possible long-term structural change after therapy (Eack et al., 2010). Results suggest a neuroprotective effect of 2 years of CR in early-course schizophrenia, against gray matter loss in regions implicated in social and non-social cognition (left parahippocampal gyrus, fusiform gyrus, and amygdala). Interestingly, authors in a more recent study observed that larger cortical surface and gray matter volume predicted a better social-cognitive response to CR (Keshavan et al., 2011).

### Change in functional connectivity after CR

Many authors suggest that CR could improve impaired functional connectivity in schizophrenia. Subramaniam et al. (2014) observed that increased right frontal activation in a verbal n-back task was correlated with activation in left frontal regions, suggesting an increased connectivity after CR. Vianin et al. (2014) described an effect of CR on left inferior frontal activation during a verbal fluency task that could indicate an improvement in language lateralization, frequently impaired in schizophrenia (Weiss et al., 2003). Penadés et al. (2013) observed the effect of 40 desk-work CR sessions for 15 schizophrenic patients, compared to patients receiving social skill training. After therapy, patients in the CR group showed a deactivation of the default mode network, and a reduction in over-activation of the fronto-parietal network (i.e. central executive network) during an n-back task that was correlated to a significant increase of the functional anisotropy in the corpus callosum. This might reflect a mechanism of functional reorganization following CR, rather than a redistribution pattern that would have produced only change in activation without functional connectivity improvements (Kelly & Garavan, 2005).

### Generalization of neural improvements on a behavioral level

In these studies, behavioral improvements are often observed after therapy, mainly in memory and executive functions. These cognitive improvements are correlated with increased activation in frontal regions (Bor et al., 2011; Haut et al., 2010; Penadés et al., 2013; Subramaniam et al., 2012, 2014) and with less gray matter loss in parahippocampal and fusiform gyrus (Eack et al., 2010). Neurobiological improvements after therapy may be mirrored by a clinical and cognitive improvement that can be observed up to 6 months after therapy (Subramaniam et al., 2014). These results are consistent with an observed behavioral efficacy of CR in literature, up to 8 months after the end of therapy, especially in verbal memory, working memory, and executive functions (McGurk et al., 2007; Wykes et al., 2003).

### Contribution of EEG and MEG studies

Parallel to MRI studies, other authors provided additional evidence of a potential neuroplastic effect of CR, using MEG and EEG techniques. Generalization of results for these studies can be restricted by a limited number of studies and the small sample of included patients. However, the differential effect of therapy in these studies can give further information about the impact of the duration and cognitive target of a CR program.

EEG and MEG studies suggest that as few as 20 hours of focused neurocognitive training can lead to focused improvements on a behavioral level, with (Popov et al., 2011, 2012) or without (Rass et al., 2012) a generalization to neurobiological measures. However, results suggest that observing widespread changes involving prefrontal cortex may necessitate longer interventions (Dale et al., 2016). Indeed, after 50 sessions of a CR program focusing on auditory working memory and verbal learning, Dale et al. (2016) found an increased M100 response, a component involved in the processing of auditory stimuli, in the transverse temporal gyrus. This improvement was correlated with a better cognitive functioning related to frontal network. Authors also observed after therapy an elevated early high gamma activity across prefronto-temporal regions, including DLPFC and secondary auditory cortex. On the contrary, Popov et al. (2011), Popov et al. (2012) did not find a similar effect on M100 response after 20 CR sessions of auditory training.

Concerning the effect of the cognitive target of CR, EEG and MEG studies provide inconsistent results. Popov et al. (2011), Popov et al. (2012) compared auditory training to a broader CR program. During an MEG paired-click task, patients had to passively listen to two similar auditory stimuli. After therapy, patients receiving auditory training had a significantly greater improvement for M50 gating ratio (i.e. decreased signal at the onset of the second stimulus, that can reflect the inhibition of redundant sensory information) that seems to reflect a better discrimination accuracy in the auditory system. Furthermore, patients that received the broader training program had a less important alpha decrease than patients in the auditory training group; this alpha decrease being positively correlated with improvement in verbal memory. Similar results were replicated in a more recent study, where the auditory training was compared to a CR program targeting social cognition (Popov et al., 2015). These results suggest that a more focused CR program targeting auditory-verbal could be more efficient on a neurobiological and a behavioral level.

On the contrary, wKariofillis et al. (2014) reported a limited effect of auditory training when compared to a broader visual training program. The auditory training showed no significantly greater improvement and no maintained results at a 2-month follow-up. After therapy, both training groups exhibited a decreased P2 event-related potential latency during an auditory selective attention task. P2 is thought to index working memory, and an increased P2 latency has been attributed to inefficient cortical processing. However, P2 latency decrease was more frontal and lasted longer after the broader visual training (Fz and FCz electrodes) than after auditory training (FCz and Cz).

Further studies comparing programs with a different duration and cognitive target are needed to clarify existing data on the effect of CR on neurocognition.

## Remediation of social cognition

Only four reports assessed the efficacy of CR on social cognition. Included studies had very small samples – ranging from 15 to 22 SCS patients – except for Popov and colleagues studies (Popov et al., 2015; Popova et al., 2014), or proposed a limited number of CR sessions targeting specifically affect recognition – ranging from 12 to 20 sessions – except for Hooker and colleagues studies (Hooker et al., 2012, 2013). Furthermore, these reports concluded to differential neural effect of affect recognition training.

A study that proposed a broad and long-lasting CR program, providing an integrative training of auditory processes and facial affect recognition, concluded to a greater activation in frontal areas implicated in complex emotion processing after training than after a positive control, during facial affect discrimination tasks (Hooker et al., 2012, 2013). In the first task, authors observed a significantly greater increase in right postcentral gyrus for positive and negative emotions compared to neutral objects. The right postcentral gyrus is known to support facial emotion recognition through internal simulation of the emotion one perceives (Adolphs, Damasio, Tranel, Cooper, & Damasio, 2000). In the second task, after therapy, patients had a significantly greater increase in bilateral amygdala for open-mouth emotions (happy, surprise, and fear) compared to baseline. Adolphs et al. (2000) suggest that amygdala is implicated in the recognition of fear and facilitates emotion identification by directing attention to distinguishing visual characteristics of the expression. Behavioral improvement in perception of emotions for all participants was correlated to an increased activation during the first task in right postcentral gyrus (emotion vs. neutral), left angular gyrus (positive vs. neutral), and left precentral gyrus (positive vs. negative). Moreover, a better performance during the second task after therapy was correlated to an increased activity in right amygdala. Interestingly, authors also observed a significant decrease in areas implicated in working memory and emotional control (left medial superior frontal, anterior cingulate, and straight gyri) for negative emotions compared to neutral objects.

In a larger sample, a report compared 20 computerized CR sessions targeting facial affect recognition to auditory training and to TAU (Popov et al., 2015; Popova et al., 2014). In two MEG studies on two subsamples of 57 patients, effect of training was assessed during a paired-click task and during a facial emotion task where faces morphed from neutral to an emotion. After auditory training only, alpha power improved during the paired-click task (alpha decrease at the onset of the second click). On the contrary, alpha power improved during the facial emotion task (alpha increase during the pre-morphing period of the task), both after facial affect recognition training and auditory training, although this improvement was greater after the social cognition training. MEG data suggest that both auditory training and facial affect recognition training targeted neural networks involved in facial discrimination (Popov et al., 2015; Popova et al., 2014).

Likewise, a cross-over EEG study on a sample of 15 patients with a history of violent offenses concluded after facial affect training to an early decrease in left parietal-temporal-occipital activation at a time window which corresponds to figural processing and encoding of faces, followed by an increased activation in the DLPFC and anterior cingulate gyrus (Luckhaus et al., 2013). This later increased activation can be linked to an emotional face processing implicating integrative and executive functions such as attribution of valence and self-referral and thus can be interpreted as a development of a more complex processing strategy after training. However, there was no change after facial affect training in P100, N170, and P250 event-related potentials associated with face perception and processing.

Finally, an fMRI study using a smaller program of 12 sessions focusing on facial affect recognition found a greater increase in activation after training than after TAU in temporal-occipital regions associated with visual information processing during a facial affect recognition task (Habel et al., 2010). Moreover, a significant correlation was found between activation increase in these areas and performance improvement in the facial affect recognition task. This activation pattern is similar to the pattern observed at baseline in the healthy volunteers sample, and is thought to be associated with the perception, association, and evaluation of emotion. In a control age discrimination task, authors observed an activation increase in different cortical and subcortical regions that are partially related to emotion processing. Identification of emotions is a complex process involving several cognitive functions, and these results seem to indicate a general improvement in the processing of visual stimuli such as faces after practice, rather than a specific effect of CR on emotion recognition.

These reports provide only limited evidence on social cognition training for schizophrenia. Results show an effect of facial affect trainings on facial affect recognition. However, it is still unclear whether the observed patterns of activation increase reflect an improvement in facial emotion discrimination itself or a general improvement in information processing.

## Conclusion

Our objective was to assess the effect of CR programs on neural processes. We selected 15 reports including 19 randomized controlled studies on 455 adult patients suffering from a schizophrenia spectrum disorder. The main limitations of this systematic review are the discrepancy in control groups as well as the small sample in several studies. Other limitations include possible risks of bias due to a partial randomization for two studies; a partial or absent blinding in the majority of the studies; a selective reporting of correlation between neurobiological and behavioral results in five reports; data substitution in three reports; patients receiving financial compensation for participation in eight reports; and financial conflict of interest in four reports. Furthermore, reporting bias may have occurred during study selection.

Included studies used various social and non-social cognition training programs, using desk-work or computerized exercises, in individual or group sessions. Neurobiological results seem independent from CR modalities. This observation is consistent with conclusions on the behavioral effect of different CR programs (McGurk et al., 2007). However, the variety of training programs and cognitive targets of CR have as a consequence an important discrepancy in the tasks used to assess neural processes. These tasks were selected to be coherent with the programs used, but their multiplicity hinders data comparability.

Interestingly, in eight reports where symptomatology was assessed before and after therapy, no significantly greater improvement was observed in the CR group when compared to control groups. These results suggest that the neural and behavioral improvements observed in these studies cannot be accounted for by an improvement in symptomatology. However, these results are not in accordance with several studies that observed an improvement in schizophrenia symptoms after CR (Wykes et al., 2003), especially negative symptoms (Bellucci, Glaberman, & Haslam, 2002). Furthermore, 7 of the 15 included reports did not assess the evolution of clinical symptoms throughout the study. Further studies taking into account symptomatology are needed in order to observe the correlation between the neural, behavioral, and clinical effects of CR in schizophrenia.

A limited number of the included studies observed functional outcome and their correlation with neural improvements. Four reports assessed global functioning and two reports assessed quality of life before and after CR, and found no significantly greater improvement in the CR condition, when compared to control conditions. These results might be explained by the fact that cognitive improvements following CR may translate to a functional level only in the long term. Indeed, one included report observed a correlation between prefrontal activation after CR and quality of life at a 6-month follow-up (Subramaniam et al., 2012, 2014). Furthermore, functional improvements have been observed in other studies 6 months after therapy (Wykes et al., 2003). Future studies observing the neural effect of CR would need to include functional assessments before and after therapy, and at follow-up. Assessing the effect of CR on quality of life, autonomy, social, and occupational functioning is of critical importance because these outcomes represent the main goals of CR in schizophrenia.

Moreover, selected studies included patients that received pharmacological treatment in parallel with CR. Despite the fact that the type of medication was controlled in reviewed studies, it might have influenced results. Indeed, there is no general consensus in the literature concerning the cognitive impact of antipsychotic medication in schizophrenia. Several studies observed a greater positive effect of atypical antipsychotic treatment compared to typical antipsychotics on learning and processing speed. On the contrary, Honey et al. (1999) concluded that substitution of risperidone for a typical antipsychotic was associated with improvement in cognitive functioning associated with frontal network over a shorter period of time, in a non-randomized study. A recent meta-analysis observed no difference in cognitive performance between medicated and drug-naïve schizophrenia patients (Fatouros-Bergman, Cervenka, Flyckt, Edman, & Farde, 2014). However, in the long-term, antipsychotic treatments might have a potential dose-dependent deleterious effect on white and gray matter volume in schizophrenia (Lett, Voineskos, Kennedy, Levine, & Daskalakis, 2014).

Interestingly, procognitive agents have been proposed, such as cholinergic and dopaminergic agents, as potential targets for the improvement of reward-related learning (Keshavan, Vinogradov, Rumsey, Sherrill, & Wagner, 2014) or glutamatergic agents as a target for memory consolidation (Michalopoulou, Lewis, Wykes, Jaeger, & Kapur, 2013). Pharmacological treatments need to be developed and tested in conjunction with cognitive training in order to potentiate therapy.

Other treatments, such as non-invasive brain stimulation methods (transcranial magnetic stimulation, transcranial direct current stimulation), have yielded promising results for the improvement of cognitive functions (Demirtas-Tatlidede, Vahabzadeh-Hagh, & Pascual-Leone, 2013). Future research needs to explore the combination of these stimulation techniques with targeted CR, in order to observe the potential effect of the modulation of neuronal excitability associated with intensive training.

Overall, present studies provide interesting conclusions on a possible neuroplastic effect of CR in schizophrenia through functional reorganization of neural networks, superior to other interventions or usual care. Specifically, CR can improve various cortical and subcortical activations, including frontal activation associated with high-level cognitive and social-cognitive functions. Further randomized controlled studies are needed to confirm or clarify existing results, in order to provide stronger evidence for a neurobiological effect of CR programs in schizophrenia spectrum disorders.

## Appendix

Search strategy: MEDLINE

01. Schizophrenia AND Remediation AND Neural (Search results: 33)

02. Schizophrenia AND Remediation AND Imaging (Search results: 25)

03. Schizophrenia AND Remediation AND Tomography (Search results: 28)

04. Schizophrenia AND Remediation AND Spectroscopy (Search results: 7)

05. Schizophrenia AND Remediation AND Electroencephalography (Search results: 5)

06. Schizophrenia AND Remediation AND Magnetoencephalography (Search results: 3)

07. Schizophrenia AND ‘Cognitive training’ AND Neural (Search results: 18)

08. Schizophrenia AND ‘Cognitive training’ AND Imaging (Search results: 12)

09. Schizophrenia AND ‘Cognitive training’ AND Tomography (Search results: 7)

10. Schizophrenia AND ‘Cognitive training’ AND Spectroscopy (Search results: 3)

11. Schizophrenia AND ‘Cognitive training’ AND Electroencephalography (Search results: 4)

12. Schizophrenia AND ‘Cognitive training’ AND Magnetoencephalography (Search results: 5)

13. Schizophrenia AND Rehabilitation AND Neural (Search results: 46)

14. Schizophrenia AND Rehabilitation AND Imaging (Search results: 72)

15. Schizophrenia AND Rehabilitation AND Tomography (Search results: 62)

16. Schizophrenia AND Rehabilitation AND Spectroscopy (Search results: 52)

17. Schizophrenia AND Rehabilitation AND Electroencephalography (Search results: 28)

18. Schizophrenia AND Rehabilitation AND Magnetoencephalography (Search results: 2)
